# Morphological Processing as We Know It: An Analytical Review of Morphological Effects in Visual Word Identification

**DOI:** 10.3389/fpsyg.2012.00232

**Published:** 2012-07-12

**Authors:** Simona Amenta, Davide Crepaldi

**Affiliations:** ^1^MoMo Lab, Department of Psychology, University of Milano-BicoccaMilan, Italy

**Keywords:** morphological processing, visual identification, response times, ERPs, eye-tracking, benchmark effects, computational models

## Abstract

The last 40 years have witnessed a growing interest in the mechanisms underlying the visual identification of complex words. A large amount of experimental data has been amassed, but although a growing number of studies are proposing explicit theoretical models for their data, no comprehensive theory has gained substantial agreement among scholars in the field. We believe that this is due, at least in part, to the presence of several controversial pieces of evidence in the literature and, consequently, to the lack of a well-defined set of experimental facts that any theory should be able to explain. With this review, we aim to delineate the state of the art in the research on the visual identification of complex words. By reviewing major empirical evidences in a number of different paradigms such as lexical decision, word naming, and masked and unmasked priming, we were able to identify a series of effects that we judge as reliable or that were consistently replicated in different experiments, along with some more controversial data, which we have tried to resolve and explain. We concentrated on behavioral and electrophysiological studies on inflected, derived, and compound words, so as to span over all types of complex words. The outcome of this work is an analytical summary of well-established facts on the most relevant morphological issues, such as regularity, morpheme position coding, family size, semantic transparency, morpheme frequency, suffix allomorphy, and productivity, morphological entropy, and morpho-orthographic parsing. In discussing this set of benchmark effects, we have drawn some methodological considerations on why contrasting evidence might have emerged, and have tried to delineate a target list for the construction of a new all-inclusive model of the visual identification of morphologically complex words.

## Paper’s Goals

Over the last 40 years, a growing number of studies have addressed the issue of morphological processing in the visual identification of complex words. While morphological effects have been consistently reported by a large number of studies, several issues are still matter of discussion, including whether processing unfolds along two different routes (e.g., Grainger and Ziegler, 2011) or just one (e.g., Crepaldi et al., 2010); whether semantics play a role since the very early processing stages (e.g., Feldman et al., 2009) or rather comes into play at a post-lexical level (e.g., Rastle et al., 2004); whether morphological analysis occurs automatically (e.g., Taft, 2004) or is context-dependent (e.g., Burani and Caramazza, 1987; Caramazza et al., 1988); and whether morphological effects need explicit morphemic representations to be accounted for (e.g., Baayen et al., 2006) or may simply emerge in the interaction between orthographic and semantic representation levels (e.g., Gonnerman et al., 2007; Baayen et al., 2011). General models of morphological processing conflict on how they deal with these issues, but the debate seems to have become somewhat inconclusive over the last decade: often new models are put forward without previous models being clearly falsified, and without an explicit comparison that could clarify whether and how the new model extends the previous ones, both in its architecture and in its explanatory power. It is thus difficult to assign credit and blame to specific aspects of competing models, with the result that our knowledge in the field does not progress in a cumulative fashion (which means, someone might argue, that it does not progress at all). Several reasons lie behind this fact, but one fundamental issue, we believe, is that several pieces of evidence are still controversial: often scholars do not argue about the best interpretation of a given fact, but about whether that fact exists at all. Stated differently, we lack a list of uncontroversial experimental effects that any general theory should be able to explain. This is the issue that we have taken up in this paper, where we review morphological effects in visual word identification, trying to disentangle those that have received strong support from those that are still weak and require more experimental work. The aim of this paper is therefore to compile a list of reliable morphological effects in visual word identification that every model should be able to explain, in the hope that this will allow an easier adjudication process between existing theories and, if necessary, the development of new theories in a cumulative, nested fashion (e.g., Grainger and Jacobs, 1996). Of course, this wish refers to general, all-inclusive models of the visual identification of complex words. In fact, the approach we are suggesting here does not exclude that specific models, more limited in scope, might be constructed to explain only a subset of the target list that we have illustrated above.

In achieving this goal, we will focus mainly on behavioral (i.e., response time based) effects for two reasons: first, in order to keep the discussion into manageable dimensions; and second, because all existing theories are defined in behavioral terms and thus can only license explicit and computationally defined predictions at this level. We also considered EEG and eye-tracking studies because their temporal resolution is fundamental in understanding the fine timing of behavioral effects, which is relevant for this special issue that is focused on the first 250 ms of visual word processing. Neuroimaging evidence will only be considered in support of behavioral data. We will also limit our review to those experimental paradigms that more directly tap onto visual word identification (such as masked priming and lexical decision), and in particular onto its early steps. Other tasks (such as, for example, word naming) or paradigms (long-SOA or cross-modal priming) will be considered only when the critical evidence can be reliably attributed to perceptual processes or to the purpose of contrasting early vs. late effects. Finally, in order to avoid any selection bias, we covered in this review any morphological effect in the visual identification of complex words that (i) we were aware of and (ii) could reliably be traced back to early processing steps. Any such effect that might be excluded from this review was only so because we failed to spot it in this vast literature.

## Morphological Effects in Visual Word Identification

### Morpheme frequency effects

The morpheme frequency effect is generally interpreted as a diagnostic index of the use of morphemes as effective processing units in complex words recognition. Such effect has been repeatedly observed in psycholinguistic research, particularly in lexical decision experiments adopting a factorial approach (i.e., modeling frequency as a two-level variable – high vs. low). For example, Taft and colleagues (Taft, 1979; Taft and Ardasinski, 2006) described both surface and stem frequency effects in derived prefixed (e.g., reproach, dissuade) and inflected (e.g., sized, parents) words. These results for inflections were later confirmed in other languages (e.g., Italian: Burani et al., 1984; French: Colé et al., 1989; Dutch: Baayen et al., 1997; Finnish: Lehtonen et al., 2007). Morpheme frequency effects for both full form and constituents have also been observed with compound words using different methodologies (mainly eye-tracking and event-related potentials; see for example, Andrews, 1986; Juhasz et al., 2003; Pollatsek and Hyönä, 2005; Vergara-Martínez et al., 2009).

Obviously, stem frequency effects can only be appropriately studied when whole-word frequency is taken under control, which typically means that this latter variable was matched between the high and low-frequency stem words being compared. By adopting this approach, however, scholars were blind for years to the fact that stem frequency might be modulated by whole-word frequency (Caramazza et al., 1988; Beauvillain, 1996; Baayen et al., 1997; Schreuder, 1997; Alegre and Gordon, 1999; Allen et al., 2003; Kuperman et al., 2008). This issue was explored by Baayen et al. (2007), who failed to find stem frequency effects in an experiment where only low-frequency words (derivations and inflections) were included. However, in a second experiment where target words spanned the entire whole-word and stem frequency range, stem frequency re-emerged as a significant factor, although modulated by whole-word frequency: stem frequency had in fact a facilitatory effect for the lowest frequency words, but an inhibitory effect for the highest frequency words. These findings emerged in an analysis of mean lexical decision times for around 8,000 words across 816 subjects as reported in the English Lexicon Project database (Balota et al., 2004), and are thus to be considered as the most reliable estimate of the stem frequency effect available to date.

Other studies have investigated whether frequency effects emerge independently of the context, or are rather contingent to, e.g., the presence of some specific type of filler items. Andrews (1986) showed that a stem frequency effect was present in the recognition of suffixed words only when compounds were also included in the experiment. A more recent study by Taft (2004) investigated word frequency effects in a lexical decision task where non-words had real vs. non-existent stems (“mirths” vs. “milphs”). This study showed that, when lexical decision is performed against nonsense stem non-words, high base-frequency words are easier to recognize than low base-frequency words as one would normally expect; but the reverse happens when lexical decision involves real-stem non-words. It does seem, then, that the overall characteristics of the entire experimental list presented to the subjects have an effect on stem frequency effects. (We point out, however, that this might not be relevant in simulation studies, where, typically, word response times are estimated as theoretical identification times with no reference to specific experimental contexts).

Some studies have gone more in depth and have tried to analyze the relationship that holds between stem and affix frequency effects. Burani and Thornton (2003), for example, demonstrated that lexical decision latencies depend on the interaction between root and suffix frequency in Italian derived words and pseudo-words. In a series of lexical decision experiments, they showed that suffixed pseudo-words (e.g., galmy, tudness) with higher frequency affixes present increased decision latencies and higher error rates, in comparison to pseudo-words with lower frequency affixes. They also showed an asymmetrical pattern for high-frequency and low-frequency roots whereby the former showed quicker and more accurate responses, while the latter did not differ from non-derived words, irrespectively of affix frequency. Results were interpreted to indicate that the main factor responsible for lexical decision performance is root frequency, with only a marginal role for affix frequency.

Finally a few studies addressed the role of affix productivity in modulating frequency effects. Bradley’s (1979) study showed a stem frequency effect only for derived words with productive endings like “-ness” or “-ment,” while derived words with less productive affixes showed only a surface frequency effect. These results were partially replicated by Vannest and Boland (1999; Experiment 1): however, the authors also report a lack of impact for root frequency when enlarging the item list to include 10 suffixes (productive: “-ship,” “-ness,” “-less,” “-hood,” “-er”; non-productive: “-ous,” “-ory,” “-ity,” “-ian,” “-ation”) instead of the three used originally in Experiment 1 (“-less,” “-ity,” and “-ation”), therefore weakening the original claim that affix productivity is a crucial factor in the modulation of frequency effects.

In sum, there is strong evidence that stem frequency influences the identification times of complex words independently of affix characteristics (e.g., frequency and productivity). Substantial evidence (although without replication as yet) is also available that stem frequency effect interacts with whole-word frequency, namely, that it is facilitatory for low-frequency words, but inhibitory for high-frequency words. Finally, evidence shows that stem frequency effects might depend on testing condition, in particular on the composition of the stimulus list.

### Morphological priming effect

Morphological priming has been so extensively observed (e.g., Forster et al., 1987; Grainger et al., 1991; Marslen-Wilson et al., 1994; Frost et al., 1997; Rastle et al., 2000; Gonnerman et al., 2007; Crepaldi et al., 2010) that it does not make any sense to ask ourselves whether it exists or not: it is an established fact that prior exposure to a morphological relative – whether briefly or for relatively long time, in the same modality or in a different one – makes the visual identification of any given word faster and more accurate. It is interesting, however, to ask which variables affect morphological priming; this is much less obvious and likely to provide constraints on morphological theories of visual word identification.

#### Frequency

When the prime is consciously visible to participants, there is evidence showing that low-frequency primes yield larger time savings than high-frequency primes, at least for derived words (Raveh, 2002). This is confirmed by data in cross-modal priming experiments, which tap on central levels of processing similarly to what long-SOA paradigms do. For example, Meunier and Segui (1999) compared high- and low-frequency spoken primes (suffixed derived words) in a visual lexical decision task, and found reliable morphological effect only for the latter. Effects of target frequency on morphological priming appear to be weaker: to the best of our knowledge, they were only reported once and with derived targets (Meunier and Segui, 1999), which is not the standard condition under which morphological priming is evaluated.

However, data from masked priming paradigms are unclear as to whether prime frequency actually matters in early stages of the word identification process. For example, McCormick et al. (2009) are clear-cut in showing no sign of interaction between prime frequency and morphological facilitation in a study on derived words. These data seem to suggest that morphological decomposition is applied to all complex words regardless of their frequency. However, Giraudo and Grainger (2000) report larger effects with high-frequency derived primes than with low-frequency derived primes. One possibility is that the different results obtained in the two studies depend on the fact that Giraudo and Grainger (2000) used a longer SOA (57 ms vs. 42 ms), but this is clearly a speculation that calls for more direct experimental support.

#### Affix and stem priming

Morphological priming is typically investigated in experiments where primes and targets share their stem (e.g., dealer-DEAL). However, most of the recent morphological models do not attribute different roles to stems and affixes in visual identification (e.g., Crepaldi et al., 2010; Baayen et al., 2011; Grainger and Ziegler, 2011) and thus we should also be able to observe affix priming.

Giraudo and Grainger (2003) did report such an effect (both with prefixes and suffixes, at least when these latter coincided with a syllable), but only in comparison with an unrelated baseline (e.g., *enjeu-ENVOL* – in English: stake-FLIGHT – vs. *biche-ENVOL* – in English: deer-FLIGHT); affixed primes never yielded significant time savings as compared to pseudo-affixed primes (e.g., *engin-ENVOL* – in English: device-FLIGHT) where the initial (or final) letter sequences did not contribute any piece of meaning to the whole-word. Giraudo and Grainger (2003) do not specify whether words in their pseudo-affixed condition were entirely decomposable into existing morphemes (similar to the English example “corner”), which might justify why they did not differ from truly affixed words. In fact, given Longtin et al.’s (2003); Rastle et al.’s (2004) and several others’ data on morpho-orthographic priming (see Rastle and Davis, 2008 for a review), a proper control condition for affix priming should be orthographically matched with the critical one, but should also contain undecomposable primes (similar to the form condition tested in those experiments, e.g., brothel-BROTH). Curiously, three affix priming studies include such a control condition, but their results are inconsistent. Chateau et al. (2002) tested prefix priming in English against an orthographically matched, monomorphemic condition (e.g., dislike-DISPROVE vs. violin-VIOLATE) and reported no significant effect. On the contrary, Dominguez et al. (2010) – working on prefixes – and Duñabeitia et al. (2008) – working on suffixes – obtained significant affix priming over and above orthographic effects. Although this might just be cross-linguistic variability, there is no obvious reason why affix priming should emerge in Spanish, but not in English. One obvious difference between these languages is that English is morphologically impoverished as compared to Spanish (perhaps in a reflection of a more general distinction between Germanic and Roman languages), but this does not seem to be related to affix saliency. More work is clearly required on this issue.

#### Semantic transparency

A series of studies, adopting a wide range of paradigms, have shown that semantics play a crucial role in modulating morphological priming in derived words (Sandra, 1990; Marslen-Wilson et al., 1994; Zwitserlood, 1994; Drews and Zwitserlood, 1995; Schreuder, 1997; Rastle et al., 2000; Longtin et al., 2003; Zwitserlood et al., 2005; Gonnerman et al., 2007; Meunier and Longtin, 2007; Rueckl and Aicher, 2008; Paterson et al., 2011). There seems to be universal agreement now that when primes are presented overtly (for at least 70 ms) or in the auditory modality, facilitation only emerges for semantically related prime-target pairs (Marslen-Wilson et al., 1994), or at least that facilitation is significantly larger with transparent than opaque pairs (Frost et al., 2000).

It has been hotly debated, however, whether this is also the case in masked priming experiments (i.e., when the prime is presented for less than 60 ms, anticipated – and sometimes followed – by a visual mask). A substantial number of studies have reported that: (i) pseudo-related pairs of words (e.g., corner-CORN) give more facilitation than what would be expected on the basis of orthographic overlap; and (ii) that this facilitation is equivalent to that yielded by truly related words (e.g., dealer-DEAL; see Rastle et al., 2000; Longtin et al., 2003; Devlin et al., 2004; Feldman et al., 2004; Rastle et al., 2004; Gold and Rastle, 2007; Lavric et al., 2007; Kazanina et al., 2008; Marslen-Wilson et al., 2008; Kazanina, 2011). However, some studies do report different results (Diependaele et al., 2005, 2009; Morris et al., 2007; Feldman et al., 2009). Some of this apparently inconsistent evidence can be reconciled on methodological grounds (see Davis and Rastle, 2010). Diependaele et al. (2005), for example, used a backward mask, mixed written and spoken targets in the same experiment, and showed three repetitions of each prime-target pairs to their participants, one of which might have been visible to some of them (SOA = 67 ms). Morris et al. (2007) also made use of a backward mask. Feldman et al. (2009) had instead several prime-target pairs in their opaque set characterized by non-systematic changes in the stem (e.g., bliss-BLISTERY, coin-COYNESS, relay-RELATION, sack-SACCADE), which was much less frequently the case in their transparent set. It seems, then, that the only genuine failure to replicate the pattern described above is reported in Diependaele et al.’s (2009) Experiment 4. A first thing to note is that, in fact, this experiment confirmed that morpho-orthographic priming is larger than form priming; where Diependaele et al.’s (2009) results depart from the streamline is in showing that transparent pairs yield larger time savings than opaque pairs. One possibility to account for this result is quite unrelated to any specific feature of Diependaele et al.’s (2009) experiment. It would just be that transparent priming is indeed numerically larger than opaque priming, but by a margin that is too small to overcome *consistently* the standard RT variability in priming experiments, and is thus typically not able to reach significance in the vast majority of the cases. This state of affairs could explain Diependaele et al.’s (2009) result on the basis of normal cross-experiment variability, which might determine occasional significant results. Related to that, Morris et al. (2007) propose that there is a significant linear trend in the effect size across transparent, opaque, and orthographic condition. It is suggested that semantic transparency effects might be graded, with semantic pairs holding the greatest effects and orthographic pairs the smallest. Clearly, this is just speculation at present; more direct experimental work is needed before one can take into question the general result that morpho-orthographic priming is (i) larger than form priming and (ii) statistically indistinguishable from transparent priming, at least in the standard masked priming paradigm.

In fact, in a recent study by Duñabeitia et al. (2011) equal facilitatory effects were reported for morpho-semantic (walker-WALK), morpho-orthographic (corner-CORN), and form-related pairs (brothel-BROTH). This experiment involved a cross-case same-different task, a variant of the Forster and Davis (1984) paradigm that was originally designed by Norris and Kinoshita (2008) to tap onto very peripheral orthographic processing. These data clearly show that morpho-orthographic effects do not depend entirely on a fixed relationship between primes and targets, but are sensitive to the task required to participants (see also Deutsch et al., 2003; Duñabeitia et al., 2007; Paterson et al., 2011); any complete model of the visual identification of complex words should be able to account for this fact.

#### Regularity

Irregularly inflected words such as “bought” are an issue for standard morphological theories. In fact, these latter consider morphemes as the smallest meaning-bearing orthographic/phonological units, thus implying a one-to-one mapping between orthography/phonology and semantics that is clearly absent in irregular words (e.g., there is no way of breaking down “bought” so that one orthographic element tells the reader what the word is about – i.e., buying something – and one orthographic element tells the reader that the word is a past tense form). This consideration has driven some scholars to propose a dual-route theory of morphology, whereby regular complex words are analyzed morphologically, whereas irregular words are stored as undivided wholes (and processed as such) in the mental lexicon (e.g., Pinker, 1991; Marslen-Wilson and Tyler, 1998; see Lavric et al., 2001 for discussion). Such proposals have implications for priming effects: because irregular words are not decomposed into their constituent morphemes, the visual identification system should fail to appreciate the morphological relationship with their stems, and so morphological priming should be absent between, e.g., “bought” and “buy,” or “drove” and “drive” (once orthography and semantics are properly controlled).

It is not clear whether this prediction is met in response time, long-lag priming experiments. Stanners et al. (1979) found that irregular past tense forms prime their base form to a lesser extent than the base form itself (Experiment 2), but because no unrelated baseline was employed, we do not know whether irregular priming was present overall. Interestingly, somewhat different results emerged with irregular derivations (e.g., “descriptive,” from “describe”), which appear to prime their base form to the same extent as regular derivations do (Stanners et al., 1979; Fowler et al., 1985). But this is a quite different issue, because, contrary to what happens in irregular inflected words, irregular derivations are still decomposable into separate and well-identified morphemes (e.g., “descriptive” into “descript-” and “-ive”), even if the stem does change in form.

In contrast, as far as masked priming is concerned, data seem to be clear-cut in showing that irregular inflected forms do facilitate the visual identification of their stems. In addition to the seminal work by Forster et al. (1987), the existence of morphological priming between irregular inflections and their base forms was documented by Kielar et al. (2008), Meunier and Marslen-Wilson (2004), and Pastizzo and Feldman (2002). Although these experiments all suffered from some methodological problems with control primes, their result were recently replicated in a study by Crepaldi et al. (2010), who provided new evidence that indeed masked irregular inflections prime their base forms, also showing that this does not depend on the system capturing morpho-orthographic sub-regularity in “lexical islands” (such as “meet,” “bleed,” “feed” and “breed,” whose past tense forms are “met,” “bled,” “fed” and “bred”; or “spend,” “send,” “bend” and “lend,” whose past tense forms are “spent,” “sent,” “bent” and “lent”): in fact, there was no significant facilitation with pseudo-irregular past tense forms (e.g., red-REED, tent-TEND).

In the ERP literature, several studies using long-lag priming report dissociation in the ways regular and irregular inflected verbs are processed (Weyerts et al., 1996; Münte et al., 1999; Rodriguez-Fornell et al., 2002). For example, Weyerts et al. (1996) showed that regular infinitives prime their inflected forms (present participle or simple present), while priming effect for irregular verbs does not reach statistical significance. Moreover ERPs patterns for regular and irregular forms diverged in waveform, peak latencies, and amplitudes. For example, regular past participle forms primed by their infinitive forms showed a P200 effect as opposed to irregular past participle forms (Weyerts et al., 1996). Interestingly, this same component was reported for repetition priming trials within the same experiment, suggesting that (i) similar mechanisms, at least in terms of their time-course, underlie repetition and regular-form priming; and (ii) regular and irregular forms processing is, at least in terms of timing, qualitatively different (Weyerts et al., 1996). In an ERP repetition priming paradigm, Münte et al. (1999) found a reduced N400 effect for regular verb pairs (stretched-STRETCH) as compared to irregular verb pairs (fought-FIGHT), which could not be linked to phonological and orthographic factors. N400 is a well-known – although highly discussed – component in the psycholinguistic literature (Kutas and Federmeier, 2011). As far as morphological processing is concerned, it has been suggested to reflect facilitated access to word stems (Morris et al., 2007). Therefore, the decreased N400 observed for regular-forms priming may indicate that regular primes are able to activate their word stems more effectively than irregular primes.

More recently however, contrasting evidence emerged in a series of studies employing ERPs (Kielar and Joanisse, 2009) and event-related magnetic fields (Stockall and Marantz, 2006). In a visual lexical decision task (SOA = 200 ms), Kielar and Joanisse (2009), compared neural responses to regular (baked-BAKE), vowel-change irregular (sang-SING), and suffixed irregulars (slept-SLEEP) prime-target pairs. The authors reported a strong N400 effect only for regular verbs seemingly indicating that regular and irregular verbs are processed differently. However, subsequent analyses differentiating early vs. late components of the N400 revealed temporal changes in the ERP pattern: while the early time interval (324–400 ms) showed the influence of formal relationship between prime and target (N400 effect for regular and ortho-phonologically overlapping pairs), the late time interval (400–476 ms) showed an effect for morphologically related pairs (regular and irregular). It appears that the difference between regular and irregular pairs might be graded and affected by the interaction of formal, semantic and phonological factors.

These results seem to confirm what was previously reported by Stockall and Marantz (2006) in a long-term priming, lexical decision, MEG study. These authors compared magnetic responses to regular and irregular prime (past participle)-target (base form) pairs, where orthographic overlap and priming direction were manipulated so as to build eight conditions tested in two separated experiments: irregular low overlap (taught/TEACH) vs. irregular high overlap (gave/GIVE) vs. identity (boil/BOIL) vs. orthographic overlap (curt/CART; Experiment 1); and irregular low overlap (teach- TAUGHT) vs. irregular high overlap (give-GAVE) vs. regular (date-DATED) vs. orthographic and semantic relation (boil-BROIL; Experiment 2). In both experiments, regular and irregular participle primed their base forms to a similar extent, with similar latencies of the M350 component – an index of root activation – in all morphologically related conditions. However it was shown that the M350 effect depended crucially on orthographic overlap and on priming direction. High orthographic overlap pairs (gave-GIVE) showed priming effects in both directions (gave-GIVE and give-GAVE); on the contrary, low orthographic overlap pairs showed an effect only when the inflected form was used as a prime (teach-TAUGHT). More interestingly, pairs that shared orthographic and semantic elements, like “boil-BROIL,” failed to show any priming effect. This data was interpreted as evidence that morphological effects cannot be explained solely on the bases of orthographic, phonological or semantic relatedness.

Taken altogether, the pattern shown in electrophysiological studies seem to suggest that regularity effects emerge only at later stages of lexical processing and that they are sensitive to pattern of sub-regularities which could be represented as the probabilistic combination of orthographic, phonological, and semantic elements (Justus et al., 2008). In conclusion, then, both behavioral and electrophysiological evidence suggests that regular and irregular inflections are processed in a similar fashion early after stimulus presentation, thus providing support for the existence of a single mechanisms operating at least during the initial stages of lexical access.

#### Free and bound stems

Morphological theories differ substantially as to whether free stems (stems that are existing words themselves; e.g., “form”) and bound stems (stems that cannot be used as words in isolation; e.g., “-mit,” as in “submit,” “permit,” and “commit”) have the same mental representation (e.g., Taft and Kougious, 2004; Crepaldi et al., 2010). It is thus not obvious whether these two types of morphemes should give rise to equivalent priming effects.

Forster and Azuma (2000) investigated this issue and discovered that bound and free stems produce equivalent facilitation, which in both cases could not be attributed solely to orthographic factors. Moreover, they found that priming with bound stems depends on affix and stem productivity (roughly, the number of different complex words where they appear). Forster and Azuma’s (2000) data were closely replicated by Pastizzo and Feldman (2004; see also Järvikivi and Niemi, 2002), using both orthographic and unrelated pairs as a baseline. In particular, these authors reported that bound stem priming correlates with the number of morphological relatives (in line with Forster and Azuma, 2000), whereas free stem priming does not.

In conclusion, there is consistent evidence that free and bound stem give rise to equivalent priming effects, even though bound stem priming seems to depend on affix and stem distributional properties.

### Transposed-letter effects and morpheme boundaries

After the seminal report by Forster et al. (1987) showed that transposed-letter (TL) primes (“anwser” for “answer”) are as effective as identity primes in facilitating visual word identification, a number of experiments have documented the so-called “jumbled word effect” (Grainger and Whitney, 2004), namely, that the word identification system tolerates imprecisions in letter position so that it tends to identify some kind of transposed-letter non-words as their corresponding words (e.g., “jugde” as “judge”; e.g., Perea and Lupker, 2003, 2004; Schoonbaert and Grainger, 2004; Lupker et al., 2008; Duñabeitia et al., 2009b). This phenomenon has crossed the morphology literature when it was shown that primes containing letter transpositions within morphemes (e.g., sunhsine) facilitate naming as much as correctly spelled primes, but primes with letter transpositions across morpheme boundaries (e.g., susnhine) do not yield any time saving as compared to substituted-letter primes (e.g., sumzhine; Christianson et al., 2005). This effect also held for pseudo-compounds (e.g., mayhem) and derived words (e.g., grinder), and was replicated by Duñabeitia et al. (2007) (i) in two more languages (Basque and Spanish), (ii) in a more standard lexical decision paradigm, and (iii) with stronger statistical support. These results were taken to show that morphological decomposition operates early, most likely before lexical identification has taken place. In line with this suggestion, Lemhöfer et al. (2011) showed that Dutch readers are quicker at recognizing compounds when their morpheme boundary is flagged by a low-frequency letter bigram (at least when the compound word was a long one). Because bigram frequency is sub-lexical information, these results strengthen the idea that morphological segmentation kicks off well before lexical identification has taken place.

However, the difference between cross-morpheme and within-morpheme TL effects does not prove to be very solid. In fact, neither Rueckl and Rimzhim (2011) in English nor Perea and Carreiras (2006) in Spanish provide converging evidence that TL effects decrease over morphemic boundaries. There are differences between these contrasting experiments that might explain inconsistencies; for example, Perea and Carreiras (2006) used compound words, whereas Duñabeitia et al. (2007) used affixed words. However, taking this into consideration does not help to reconcile the existing evidence into a coherent and clear frame. For example, on the basis of the Spanish data one might suggest that morphological modulation of TL effects emerges in affixed, but not in compound words. This proposal is contradicted by the English data, where compound words generate interaction between morphemic boundaries and TL effects (Christianson et al., 2005), but mixed results were obtained on affixed words (Christianson et al., 2005, and Rueckl and Rimzhim, 2011). Clearly, more work is necessary before it will be possible to take a stand on this issue.

### Morphological effects in non-word processing

It has long been debated whether the visual word identification system gets access to morphological information *before* lexical identification (readers would identify morphemes first, and then words; e.g., Taft, 1994), or rather *upon* lexical identification (readers would identify words first, and then become aware of their morphological structure; e.g., Giraudo and Grainger, 2001). Crucial for this debate is what happens to non-words that are morphologically structured (e.g., shootment), for which, clearly, lexical identification never occurs; observing morphological effects on this type of stimuli would thus be strong evidence for pre-lexical morphological processing.

In a seminal study, Taft and Forster (1975) reported that non-words composed of an existing prefix and an existing stem (dejeuvenate) are slower to be rejected than non-words composed of an existing prefix and a non-stem (depertoire). In a similar way, compound non-words where the first constituent is a word (footmilge) take longer to be rejected as non-word in comparison to compound non-words where the second constituent is a word (thernlow; Taft and Forster, 1976). This pattern was more recently confirmed by an Italian ERP study using a lexical decision task to compare neural responses to compound and simple words and non-words (El Yagoubi et al., 2008). This study provided clear evidence that non-words composed by an existing word and a non-word (*drillococco* – in English: drilecoconut) elicited a more negative N400 than non-words composed by two existing words (*spadapesce* – in English: fishsword), thus suggesting that existing stems embedded in non-words might trigger lexical access, mitigating the difference between words and non-words (see also, Fiorentino and Poeppel, 2007).

This morpheme interference effect was then generalized to the inflectional domain and to derived, pseudo-suffixed words (although with more controversial data). Caramazza et al. (1988) showed that pseudo-inflected Italian non-words (“*cantevi,”* similar to the English “buyed”) were rejected more slowly than non-words made up of a real-stem and a non-suffix (“*cantovi,”* similar to “buyel”), a non-stem and an existing suffix (“*canzevi*,” similar to “beyed”), and a non-stem and a non-suffix (“*canzovi*,” similar to “beyel”; see also Leinonen et al., 2009, Experiment 1, for convergent ERP data in Finnish). Again testing Italian readers, Burani et al. (1997) reported that suffixed non-words (e.g., “*vetrezza*,” lit. “glassness”) are more difficult to reject in a lexical decision task than non-words composed of an existing stem and a non-suffix (e.g., “*vetralle*,” similar to “glassmilp” in English), but only when the final part of the word is a frequent word-ending. In apparent contrast with these data, Burani et al. (2002) obtained no difference between rejection times on suffixed non-words (e.g., “*donnista*,” lit. “womanist”) and rejection times on orthographically controlled non-words that did not contain any morpheme (e.g., “*dennosto*,” similar to “wemanost” in English); a difference between the two conditions, however, emerged in the analysis of the error rates. More recently, Crepaldi et al. (2010) investigated the same issue with English material, and confirmed the pattern of results obtained by Burani et al. (1997), i.e., that suffixed non-words (e.g., gasful) take longer to be rejected than orthographic controls with non-morphological endings (e.g., gasfil). In consideration of the fact that similar morpheme interference effects have also been reported for pseudo-compounds (e.g., “pipemeal”; Taft, 1985), we would conclude that, even if some inconsistent result does appear in the literature, there is sufficient evidence to hold that morphologically structured non-words are more difficult to reject than appropriately matched orthographic controls. Incidentally, this pattern of results fits well with the ERP evidence provided by McKinnon et al. (2003), who showed similar brain responses for real words and morphologically structured non-words, thus indicating similar processes for the two types of stimuli.

Interestingly, the importance of these data on the role of morphemes in non-word processing was further strengthened by the report of masked morphological priming with non-word primes. For example, Meunier and Longtin (2007) found that response times on stem words such as “sport” are made faster by morphologically related non-word primes, such as “sportation.” This was shown to be independent from whether non-words were semantically interpretable (e.g., quickify vs. sportation), or designed to be synonymous with existing words (e.g., “brightment,” which most people would consider to mean the same thing as “brightness”). These data were confirmed in English by McCormick et al. (2009).

On the whole, then, it is clear that non-words with a morphological structure are analyzed in terms of their morphemes, thus questioning seriously any theory that suggests morphological processing to kick off upon lexical identification.

### Morpheme position effects

Capitalizing on the morpheme interference effects described in the previous paragraph, scholars have recently started to investigate how morpheme position is coded in the visual identification system. This is an important issue from a theoretical point of view, because no morphological model proposed so far has taken a stand in this respect.

Crepaldi et al. (2010) have reported evidence that suffix position coding is locked to word-final positions (or at least to post-stem positions). These authors showed that, while “shootment” is slower to be rejected than its orthographic control “shootmant” (see Burani et al., 1997), “mentshoot” and “mantshoot” are equally difficult; this was taken as a proof that “ment” is not identified as a suffix in “mentshoot” (i.e., in word-initial position), which is evidence that its representation in the visual identification system is position-specific.

More work was carried out on free stem position coding, i.e., on constituent coding in compounds (and pseudo-compounds; e.g., Taft, 1985; Taft et al., 1999; Shoolman and Andrews, 2003; Duñabeitia et al., 2009a). The evidence accumulated so far is suggestive of two facts, namely, (i) that free stems are coded in a position-independent fashion (i.e., they are identified even when they lie in unusual positions, as for “honey” and “moon” in “moonhoney”), and (ii) that their position is coded flexibly, so that, e.g., “moon” in “moonhoney” drives some activation to the word “honeymoon,” even if the position of the stem in the stimulus (word-initial) and in the target word (word-final) do not match. These conclusions are based on the observation that reversed compounds (e.g., “doorback”) seem to take longer to be rejected than control pseudo-compounds (e.g., pipemeal; Taft, 1985; Taft et al., 1999; Shoolman and Andrews, 2003), and that constituent priming occurs in a cross-position fashion (e.g., “hang*over*” primes “*over*come”; Duñabeitia et al., 2009a). A word of caution is necessary here however, because this evidence comes either from experiments where morpheme position was not the main issue, and thus some methodological details were not clear of problems (Taft, 1985; Shoolman and Andrews, 2003). More direct evidence on this issue would be desirable.

### Stem homographs effect

Stem homographs are complex words with stems that are orthographically identical, but semantically and – theoretical linguists might say – morphologically unrelated. Examples of these words abound in Neo-Latin languages such as Italian (“*colp-a*,” “fault,” and “*colp-o*,” “stroke”) and Spanish (“*mor-os*,” “moors,” and “*mor-ir*,” “to die”), and have been quite extensively studied in the nineties (Laudanna et al., 1989, 1992; Allen and Badecker, 1999, 2002; Badecker and Allen, 2002). This type of words is interesting because of its close relationship with morpho-orthographic effects: stem homographs share in fact an orthographically defined stem (just as “corner” and “corn” do) and are entirely decomposable into existing morphemes.

In two very early studies, Laudanna et al. (1989, 1992) reported an inhibitory effect by stem homographs in Italian, which was later confirmed by Allen and Badecker (1999) in Spanish (see also Barber et al., 2002; Carreiras et al., 2005; and Domínguez et al., 2004 for converging eye-tracking and ERP evidence). These were all long-SOA priming studies that allowed participants to fully process primes; it is not surprisingly, then, that stem allomorphs inhibit each other (most likely because of competition at the semantic level). In line with this consideration, and with the more recent literature on morpho-orthographic segmentation, stem homographs were found to facilitate each other in a masked priming experiment (Badecker and Allen, 2002), where instead participants were prevented from processing primes up to the semantic level.

Interestingly, Domínguez et al. (2004), using event-related potentials, were able to trace the time-course of the stem-inhibition effect reported in long-SOA priming studies, and to disentangle the effect from orthographic confounds. In a lexical decision, long-lag priming experiment (SOA = 200 ms), the authors reported an early (250–350 ms time window) overlap of stem homographic (*foco-FOCA* – in English: floodlight-SEAL) and morphological (*hijo-HIJA* – in English: son-DAUGHTER) priming waves. However, starting from 350 ms, the two wave patterns started to differ, with stem homographs producing a delayed N400 effect. Interestingly, orthographic pairs (*rasa-RANA*- in English: flat-FROG) did not produce any facilitative effect in the 250–350 ms time window, but later showed a N400 effect comparable to the one elicited by unrelated pairs.

The evidence available thus indicates that at early steps in lexical access, stem homographs have access to a common representation; however, at a later stage of semantic processing, they seem to activate two different and competing mental representations, thus resulting in the inhibitory effect commonly observed in long-SOA priming studies.

### Paradigmatic effects: Family size and entropy

Two morphological effects were described over the last 15 years in the lexical decision task that do not refer to the morphological structure of the word-to-be-processed itself, but rather to the morphological family where that word belongs. This refers to the family size effect (e.g., Schreuder, 1997; Bertram et al., 2000a; Pylkkänën et al., 2004; Juhasz and Berkowitz, 2011), whereby words with more morphological relatives are processed faster than words with a few morphological relatives, and to entropy effects (e.g., Moscoso del Prado Martín et al., 2004), whereby words with equally frequent morphological relatives are processed faster than words whose morphological family is characterized by a few very dominant members. These effects were observed in the processing of both simple (e.g., Baayen et al., 2006) and complex words (e.g., Bertram et al., 2000a; Kuperman et al., 2010; Baayen et al., 2011), and were also shown to hold independently of other, more established, lexical variables, such as cumulative family frequency, surface frequency, and neighborhood density (Schreuder, 1997). Interestingly, Schreuder (1997) also showed how family size effect progressively decreases with priming demasking, thus indicating that the effect is most likely semantic in nature, and emerges at a later, post-identification stage of lexical processing (see also De Jong et al., 2000). This effect is also one of the very few which have been shown to hold across different language families (Indo-European vs. Semitic; Moscoso del Prado Martín et al., 2005), which strengthens its reliability.

### Affix distributional properties: Allomorphy and productivity

Other factors that might affect how a morphologically complex word is processed are connected to the distributional properties of its constituent morphemes, in particular, allomorphy and productivity. These features have been suggested to concur to determine affix salience (Schreuder and Baayen, 1994; Laudanna and Burani, 1995; Burani et al., 1997; Järvikivi et al., 2006), and, in turn, to affect the probability of an affix to be activated as a specific processing unit during word recognition (Allen and Badecker, 1999;Bertram et al., 1999, 2000b), thus balancing storage and parsing processes for what concerns both inflected and derived words (Bertram et al., 1999, 2000b).

In lexical decision studies, words including affixes with several allomorphs resulted in longer latencies (Laudanna and Burani, 1995; Järvikivi et al., 2006). Moreover, Allen and Badecker (1999) showed an inhibitory effect for Spanish targets that were preceded by primes allomorphically related to their homographs (e.g., “*cierra*,” (he) closes, whose stem, “*cierr-*,” is an allomorph of the main stem of the verb “to close”, “*cerr-*”, inhibited “*cerro*”, hill) (see Linares et al., 2006, Experiment 2, for convergent ERP results).

Affix productivity has been defined in several different ways, which makes quite difficult to establish its role in the visual identification of complex words. Laudanna et al. (1994) used as an index of productivity the proportion between the number of words in which a given affix appeared as such (e.g., “driver” for “-er”) and the number of words in which the same affix did not play any morphological role (e.g., “corner” for “er”). Adopting this definition, they found that non-words including productive affixes were harder to reject than non-words including non-productive affixes. Investigating Finnish and Dutch, Bertram et al. (1999, 2000b) came to somewhat different conclusions. Without giving any exact definition of productivity, but using affixes supposedly lying at the opposite extremes of its distribution, Bertram and colleagues conclude that productivity does not have a well-identifiable effect on processing times, but interacts with word formation type (derivation vs. inflection) and affixal homonymy (an interaction that has received no independent confirmation). Finally, Plag and Baayen (2009) report effects of the number of words including any given affix on word naming times, but not on lexical decision times, again in apparent contrast with what found by Laudanna et al. (1994). All in all, there does not seem to be clear evidence to hold that productivity, however defined, influences word identification times.

### Inflection, derivation, and composition

In closing this review, we turn our attention to an issue that is cause of pain to many scholars in the field, namely, that the literature on inflection, derivation, and (in particular) compounding appears to be somewhat disconnected, perhaps under the assumption that these morphological processes are too different from each other to be reciprocally informative.

Indeed inflection, derivation and composition are very different morphological processes. Inflectional processes do not result in a new lexical entity, while derivation and composition always do (Kurylowicz, 1964). Inflection never involves a change in grammatical class, which is instead most frequently the case in derivational processes (e.g., deal-dealer). Inflection generally preserves the meaning of the stem, whereas this is not always the case in derivation (e.g., angel-angelic; Aronoff, 1976). Again, whereas inflection implies a consistent and predictable semantic change (“table” and “tables” entertain the same semantic relationship that holds between “idea” and “ideas” or “cat” and “cats”), this is much less the case in the derivational domain (e.g., while a “gardener” is a professional who takes care of gardens, a “juicer” is a kitchen appliance) and in compounding (“honey” has very different meanings in “honeycomb” and “honeymoon”).

Most of these differences are based on syntactic and semantic processes, which are unlikely to be in action very early after stimulus presentation. In fact, we would claim that, at least for what concerns the more peripheral stages of visual word identification, there is not much psycholinguistic evidence suggesting different processing of inflected, derived and compound words.

In support of this statement, Leinonen et al. (2008) and Álvareza et al. (2011) reported that ERPs patterns for inflected and derived words start to diverge around the 300–450 ms time window, with effects spilling over to the 450–550 ms time window for inflected words, thus suggesting that differences between inflection and derivation is apparent only at a later stage of lexical processing, when semantics is more likely to come into play.

Support in this direction also comes from a paper by Raveh (2002), where – in a rare direct comparison between derivational and inflectional priming – inflected and derived words yielded equivalent time savings in the identification of their stems at a brief SOA (50 ms), whereas a difference emerged later on (inflected words gave more priming at SOAs of 150 ms and 250 ms).

Substantial similarity between morphological effects with derived and compound words also emerges when considering morpho-orthographic segmentation. The vast majority of this literature has investigated derived and pseudo-derived words (see above), but in a recent paper Fiorentino and Fund-Reznicek (2009) reported significant and equivalent masked priming effects for transparent (teacup-TEA) and opaque compounds (honeymoon-HONEY, carpet-CAR), as compared to orthographic, non-morphological controls (penguin-PEN). The effect held for both initial and final constituent word priming (flagpole-FLAG vs. classroom-ROOM), and clearly mirrors what has been reported for derived words, thus suggesting that the early morpho-orthographic segmentation proposed by Rastle et al. (2004) generalizes to all types of morphologically complex words.

Perhaps even more strikingly, data gathered on inflected and compound words are closely similar for what concerns the rejection time of morphologically structured non-words in lexical decision tasks. In fact, it has been documented, for both pseudo-inflected and pseudo-compound non-words, that non-words made up entirely by non-existing morphemes (e.g., “iblish” and “thrimnade”) or by a non-existing first element and an existing second morpheme (e.g., “ibvive” and “flurbpair”) are easier to reject than non-words made up of a real morpheme as a first element followed by a non-existing second element (e.g., “inlish” and “spellcung”). In turns, these latter non-words are easier to judge than non-words entirely made up of real morphemes (e.g., “invive” and “toastpull”; see Taft and Forster, 1975; Lima and Pollatsek, 1983; Taft et al., 1986; and Monsell, 1985).

Clearly, this evidence is far from suggesting that the visual identification system processes inflected, derived and compound words in exactly the same way. However, it does suggest that at least some (peripheral) processing steps are common to all types of complex words and, more generally, that there should be a more tight integration between the literature on inflected, derived and compound words.

## The Target List

In this paper we reviewed the behavioral literature on the visual identification of complex words with the aim of building a list of established facts that might help in adjudicating between existing theories, and eventually in developing a comprehensive computational model of how complex words get identified by the visual system.

The list should include these effects:

- Stem frequency has a facilitatory effect on low-frequency words, and an inhibitory effect on high-frequency words;
- Non-words that are morphologically structured are more difficult to reject in lexical decision, no matter whether they are pseudo-prefixed, pseudo-suffixed, or pseudo-inflected;
- Non-words that are morphologically structured, but that contain a suffix at their onset are as easy to reject in lexical decision than orthographic control non-words;
- Words with larger family size are identified more quickly;
- Words with higher entropy are identified more quickly;
- Words including affixes with several allomorphs yield longer lexical decision times;

In unmasked priming:

- Low-frequency complex words yield time savings on the identification of their stems more than high-frequency complex words do;
- Morphological effects emerge only for semantically related prime-target pairs;
- Stem homographs (and their allomorphs) inhibit each other;
- Inflectional priming is larger than derivational priming;

In masked priming:

- Morphological effects emerge to the same extent for transparent and opaque prime-target pairs (but when masked priming is employed in tasks other than lexical decision, facilitation might not emerge at all for both transparent and opaque pairs);
- Morphologically structured non-words facilitate the identification of words sharing their stem;
- Irregularly inflected words prime their stems;
- Both free and bound stems determine time savings when they are shared between primes and targets;
- Bound stem priming is proportional to stem productivity (i.e., the number of different complex words where they appear);
- Stem homographs facilitate each other;
- Inflectional and derivational priming are equivalent;

From a theoretical point of view, it is not easy to see in a glimpse whether these effects speak clearly against or in favor of any existing theory. Surely, morphological effects in non-words exclude the possibility that morphological information only comes into play after lexical identification. For what concerns the other big dichotomies illustrated at the beginning of the paper (e.g., one vs. dual-route models; PDP vs. localist models), there is no clear indication popping out. This is exactly where computational modeling comes as a useful tool; in fact, by implementing theories in a computer program it becomes easier to understand unequivocally which model survives confrontation with the data (in particular for what concerns the simulation of several effects with the same system settings), and which does not.

Obviously, this list is by no means definitive (new evidence is continuously arising on what seems to be a hotly debated topic), nor necessarily complete. We made all our efforts to ensure that we covered all the relevant data, but with such a huge amount of evidence amassed over the last 40 years, it is possible that we have missed some important results. We encourage anyone to flag possible gaps, also taking advantage of the brilliant “Comment” tool made available upon the open-access policy adopted by this Journal.

The main point that we want to make with this paper, however, is not about the list *per se*; rather, we hope that having a list of benchmark effects will help the field to move forward in a more cumulative and cooperative fashion. In the spirit of the nested modeling principle put forward more than a decade ago in the related field of reading aloud (Grainger and Jacobs, 1996), we hope that in the near future (i) existing models will confront on the basis of their ability to account for these (or other) benchmark effects; (ii) credit and blame will be assigned to specific parts of each theory for their successes and failures in this attempt; (iii) in proposing any new theory, substantial effort will be spent in explaining how the new theory relates with its predecessors, how it extends them, why it does that in the way that it does, which new effects it is able to explain that its predecessors were not able to explain, and which effects it is still not able to explain that were also outside the grasp of its predecessors.

## Conflict of Interest Statement

The authors declare that the research was conducted in the absence of any commercial or financial relationships that could be construed as a potential conflict of interest.
